# Depressive Symptom Clusters in Relation to Body Weight Status: Results From Two Large European Multicenter Studies

**DOI:** 10.3389/fpsyt.2019.00858

**Published:** 2019-11-21

**Authors:** Sabrina Baldofski, Nicole Mauche, Ezgi Dogan-Sander, Mariska Bot, Ingeborg A. Brouwer, Nadine P. G. Paans, Mieke Cabout, Margarita Gili, Gerard van Grootheest, Ulrich Hegerl, Matthew Owens, Miquel Roca, Marjolein Visser, Ed Watkins, Brenda W. J. H. Penninx, Elisabeth Kohls

**Affiliations:** ^1^Department of Psychiatry and Psychotherapy, Medical Faculty, University Leipzig, Leipzig, Germany; ^2^Department of Psychiatry, Amsterdam Public Health Research Institute, Amsterdam UMC, Amsterdam, Netherlands; ^3^Department of Health Sciences, Faculty of Science, Amsterdam Public Health Research Institute, Vrije Universiteit Amsterdam, Amsterdam, Netherlands; ^4^Institut Universitari d’ Investigació en Ciències de la Salut (IUNICS/IDISPA), Rediapp, University of Balearic Islands, Palma de Mallorca, Spain; ^5^Depression Research Centre, German Depression Foundation, Leipzig, Germany; ^6^Senckenberg-Professorship, Department of Psychiatry, Psychosomatic Medicine and Psychotherapy, University Hospital Frankfurt–Goethe University, Frankfurt am Main, Germany; ^7^Department of Psychology, College of Life and Environmental Sciences, University of Exeter, Exeter, United Kingdom

**Keywords:** depression, depressive symptoms, obesity, overweight, body mass index

## Abstract

**Background:** There is strong evidence for a bidirectional association between depression and obesity. Several biological, psychological, and behavior-related factors may influence this complex association. Clinical impression and preliminary evidence suggest that patients with a diagnosis of major depressive disorder may endorse very different depressive symptom patterns depending on their body weight status. Until now, little is known about potential differences in depressive symptoms in relation to body weight status.

**Objective:** The aim of this analysis is the investigation of potential differences in depressive symptom clusters (mood symptoms, somatic/vegetative symptoms, and cognitive symptoms) in relation to body weight status.

**Methods**: Cross-sectional baseline data were derived from two large European multicenter studies: the MooDFOOD Trial and the NESDA cohort study, including persons with overweight and obesity and normal weight reporting subthreshold depressive symptoms (assessment *via* Inventory of Depressive Symptomatology Self-Report, IDS-SR30). Different measures for body weight status [waist-to-hip ratio (WHR) and body mass index (BMI)] were examined. Propensity score matching was performed and multiple linear regression analyses were conducted.

**Results**: A total of *n* = 504 individuals (73.0% women) were analyzed. Results show that more somatic/vegetative depressive symptoms, such as pain, change in appetite and weight, gastrointestinal symptoms, and arousal-related symptoms, were significantly associated with both a higher BMI and higher WHR, respectively. In addition, being male and older age were significantly associated with higher WHR. Mood and cognitive depressive symptoms did not yield significant associations for both body weight status measures.

**Conclusions:** Somatic/vegetative symptoms and not mood and cognitive symptoms of depression are associated with body weight status. Thus, the results support previous findings of heterogeneous depressive symptoms in relation to body weight status. In addition to BMI, other body weight status measures for obesity should be taken into account in future studies.

**Clinical Trial Registration:** www.ClinicalTrials.gov, identifier NCT02529423.

## Introduction

Major depressive disorder (MDD) is among the most prevalent and disabling mental disorders, one of the leading causes of disability worldwide, and a major contributor to the overall global burden of disease (1). One of the most prevalent somatic comorbidities of MDD is obesity (2, 3). Several studies have provided evidence for a bidirectional link between depression and obesity in a way that the presence of one is increasing the risk of developing the other (3–6). Further, as stated by Milaneschi et al. (3), there is strong reason to believe that these conditions are interconnected through a vicious, mutually reinforcing cycle of adverse physiological adaptations.

Several factors, such as biological, psychological, and behavior-related ones, may influence this complex association between depression and obesity (e.g., 7, 9–12). Moreover, depression and obesity are two major risk factors of negative health outcomes (10, 13, 14).

The inter-individual heterogeneity of depressive symptoms in different patients with MDD grants to a greater variability in its association with obesity. This association seems to be stronger in certain subgroups of patients (3, 6, 15, 16); for example, the association between depression and obesity is found to be stronger for abdominal obesity, and in some studies and certain populations, it was found to be inverse or even absent (17–19). Several (e.g., genetic and inflammatory) factors and mechanisms are being discussed in the international literature to date (3, 10, 20–22). Genetic factors influence likewise depression and obesity, with additive effects explaining the phenotypic variation for both depression and body mass index (BMI) (3, 23, 24). To sum up, there is emerging evidence that the relationship between depression and obesity has its origins in partially overlapping genetic bases (3).

The clinical impression also supports the hypothesis that patients with the same MDD diagnosis may endorse very different symptom patterns depending on their body weight status (3). For example, obesity seems to be associated with fatigue in the sense of tiredness (lack of drive and sleepiness), which may result from somatic reactions to the condition of obesity, whereas typical depression is associated with inhibition of drive and long sleep latencies (25–27).

In the past decades, quite some efforts have been made to determine and establish different depressive symptom patterns or clusters and examine their influence on various health-related outcomes. In most of these studies, depressive symptom patterns or clusters were distinguished as follows: a) two symptom clusters: cognitive-affective (e.g., pessimism, guilt, and self-dislike) and somatic-affective (e.g., insomnia, fatigue, and work difficulty) depressive symptoms (28, 29) or b) three symptom clusters: cognition/mood, anxiety/arousal, and vegetative symptoms (or sleep) (30, 31). However, other distinctions were also proposed, such as a differentiation between atypical and melancholic depressive subtypes (32–34).

As mentioned above, a growing body of evidence indicates that abdominal obesity is a more important risk factor of MDD than general obesity (35). It seems to be the key mediator in the relationship between obesity and depression (36). Results indicate a positive association between abdominal fat distribution [measured by waist-to-hip ratio (WHR)] and prevalence of depression, and further that abdominal obesity is a risk factor of depression independently of general obesity (measured by BMI). Nevertheless, some epidemiological studies report no association between unfavorable waist circumference and mental disorders (in particular depression) (17). Considering these differential associations, it seems beneficial to take into account different measures for body weight status, not only BMI (37).

However, only a few studies have addressed anthropometric measures in relation to different depressive symptoms so far (38), with equivocal results: one study reported that BMI was associated with both cognitive-affective and somatic-affective depressive symptom patterns of the Beck Depression Inventory I (BDI-I), whereas waist circumference and WHR were only associated with somatic-affective, but not cognitive-affective symptoms (39). Another study reported as a secondary result that BMI at baseline was significantly correlated with improvement in neurovegetative and cognitive symptoms of depression (2). It has also been shown in a population-based study that only the somatic, not the cognitive-affective, symptoms of depression are positively associated with anthropometric measures of obesity (35).

The aim of this study is the investigation of self-rated depressive symptoms (assessed with the Inventory of Depressive Symptomatology Self Report, IDS-SR30 clusters: mood symptoms, somatic/vegetative symptoms, and cognitive symptoms) in relation to body weight status in persons with overweight and obesity and normal-weight individuals reporting subthreshold depressive symptoms. Different measures for body weight status (WHR and BMI) will be examined.

## Methods

### Study Design

This publication includes baseline data from two large European multicenter studies: the MooDFOOD Trial (“Multi-country cOllaborative project on the role of Diet, Food-related behavior, and Obesity in the prevention of Depression”) and the NESDA Study (“The Netherlands Study of Depression and Anxiety”), respectively.

The MooDFOOD Trial for the prevention of depression in individuals with overweight and obesity reporting subclinical depressive symptoms is a 2 × 2 factorial randomized controlled trial. It was carried out between July 2015 and October 2017 in four European countries (Germany, Spain, United Kingdom, and the Netherlands). For full details of trial design and protocol see Roca et al. (40), and for primary outcome results see Bot et al. (41). The trial was approved by the Human Research Ethics Boards of all four study sites. All participants provided written informed consent prior to participation.

The NESDA Study is an ongoing multicenter, longitudinal, naturalistic cohort study examining the 9-year course and consequences of depressive and anxiety disorders (for details of study design and protocol, see 42). Baseline assessments of NESDA took place between 2004 and 2007. The study includes persons with a current or lifetime diagnosis of depression and/or anxiety disorder, persons being at risk for these disorders because of a family history or subthreshold depressive or anxiety symptoms, and healthy controls, respectively. Ethics approval was provided by the local review boards of all study sites and written informed consent was obtained from all participants prior to participation.

For this publication, baseline data from both the MooDFOOD Trial (total *N* = 1,025) and the NESDA Study (total *N* = 2,981) were combined to provide a sample with a wide range of body weight status. To this end, only the data of normal-weight participants from the NESDA Study were used to provide a matching sample to the MooDFOOD data of individuals with overweight and obesity (see below for details on propensity score matching procedure).

### Recruitment and Eligibility Criteria

Participants for the MooDFOOD Trial were recruited from the general population *via* websites, advertisements, and press releases as well as *via* other studies conducted at the study sites and mailings to registered subjects in the general practice setting or in city registers. Inclusion criteria were age 18-75 years, BMI in the range of 25–40 kg/m^2^, and subclinical depressive symptoms as operationalized by a Patient Health Questionnaire (PHQ-9) score of ≥5 (43). Main exclusion criteria included a current (including the past 6 months) MDD episode according to the Diagnostic and Statistical Manual of Mental Disorders (DSM-IV; (44), as assessed by the Mini-International Neuropsychiatric Interview (MINI 5.0; 45), current use of antidepressants, current eating disorder or a severe life-threatening physical disease (e.g., cancer), and a history of psychosis, bipolar disorder, substance dependence, or another severe psychiatric disorder (40). All eligible participants were invited for a baseline interview, physical measurements, and blood sampling conducted by trained research assistants/nurses and the completion of self-report questionnaires. Follow-up assessments took place at 3, 6, and 12 months.

Participants for the NESDA Study were recruited from community samples, primary care practices, and mental health organizations, in order to represent diverse settings and developmental stages of psychopathology. Participants aged 18–65 years were included in the study. Further inclusion criteria were a current (including the past 6 months) or lifetime diagnosis of depression (minor or major depression, dysthymia) or anxiety disorders according to the DSM-IV, as assessed by the Composite International Diagnostic Interview (CIDI, WHO version 2.1; 46). In addition, persons with a family history of depression or anxiety disorders and with subclinical depressive or anxiety symptoms were included, as well as healthy controls without any depressive or anxiety symptoms. Main exclusion criteria were psychosis, obsessive compulsive disorder, bipolar disorder, severe addiction, and a history of stroke (42). Eligible participants were invited for a baseline assessment including clinical interviews and physical measurements conducted by trained research assistants and the completion of self-report questionnaires. Follow-up assessments took place every 2 years after baseline and are currently ongoing.

### Propensity Score Matching and Study Sample

The baseline data of the MooDFOOD Trial included *N* = 1,025 participants in total. To match these data with the data of the NESDA Study, *n* = 35 participants had to be excluded from the dataset [*n* = 14 with normal body weight and *n* = 21 with history of stroke (exclusion criterion in the NESDA sample)], resulting in a dataset of *n* = 990 participants from the MooDFOOD Trial.

Baseline data of the NESDA Study included *N* = 2,981 participants. Of these, *n* = 2,544 had to be excluded during data preparation for the following reasons: *n* = 1,481 not normal body weight, *n* = 540 with current MDD (as this was an exclusion criterion in MooDFOOD), *n* = 113 with antidepressant medication, *n* = 94 with alcohol dependence, and *n* = 316 with anxiety disorders. Thus, the final selected sample out of the NESDA Study comprised *n* = 437.

To obtain a final sample with a wide range of body weight status, including persons with overweight and obesity from the MooDFOOD Trial as well as normal-weight persons from the NESDA Study, propensity score matching was performed (47). Based on their propensity scores calculated by logistic regression (nearest neighbor matching algorithm, caliper 0.2), samples were matched according to sex, age, and distribution of IDS scores. The final matched sample comprised *n* = 504 participants (*n* = 252 from the MooDFOOD Trial and *n* = 252 from the NESDA Study).

### Measures

*Depressive Symptoms*. In both the MooDFOOD Trial and the NESDA Study, the 30-item Inventory of Depressive Symptomatology Self-Report (IDS-SR30; 30, 48) was administered to assess depressive symptoms (0 = *no problems* to 3 = *severe problems*). The two separate items on weight loss and weight gain were recoded into a single variable. The two items on increased and decreased appetite, respectively, were recoded similarly, resulting in a total of 28 items for the IDS (9, 31). A total sum score was calculated (range, 0–84), with higher scores indicating higher levels of depressive symptomatology. In accordance with previous studies, individual symptoms were categorized into three depressive symptom clusters (deductively defined): mood symptoms, cognitive symptoms, and somatic/vegetative symptoms (9, 49, 50). A sum score was calculated for each cluster (mood symptoms: 10 items, range 0–30; cognitive symptoms: 4 items, range 0–12; somatic/vegetative symptoms: 14 items, range 0–42; see **Supplementary Table**).

*Body Weight Status*. Body weight, height, and waist and hip circumference were measured objectively according to written, standardized protocols (identical measurement in both subsamples). BMI (in kilograms per square meter) and WHR (waist circumference divided by hip circumference) were calculated.

*Presence of Lifetime MDD*. The presence of a lifetime diagnosis of MDD according to the DSM-IV (44) was assessed in all study participants using clinical interviews, specifically the MINI 5.0 (45) in the MooDFOOD Trial and the CIDI [WHO version 2.1 (46)] in the NESDA Study.

*Demographic Variables*. Sex and age were assessed in the baseline interview in both, MooDFOOD and NESDA.

All applied interview and questionnaire instruments demonstrated good reliability and validity (51–53). The IDS showed good internal consistency with Cronbach’s *α* = .80.

### Statistical Analysis

Statistical analyses were performed using IBM SPSS Statistics version 24.0. Differences in sample characteristics between participants from MooDFOOD and NESDA, respectively, were examined using general linear model analyses for continuous variables (age, BMI, WHR, and IDS scores) and *χ* ^2^ tests for categorical variables (sex and prevalence of lifetime MDD). To analyze the relationship between depressive symptoms and body weight status, two separate multiple linear regression analyses were computed with the anthropometric measure (BMI and WHR) as the continuous dependent variable and the depressive symptoms (clusters: mood symptoms, cognitive symptoms, and somatic/vegetative symptoms) and sociodemographic variables (sex and age) as the independent variables. Tests for assumption of collinearity of the independent variables showed that multicollinearity was not a concern. The independent variables were entered into the models simultaneously. Effect size of prediction was evaluated according to Cohen (54), *R* ^2^: small, .01; medium, .09; large, .25. A two-tailed *α* = 0.05 was applied to all statistical testing.

## Results

### Sample Characteristics

Participants from MooDFOOD (*n* = 252) and NESDA (*n* = 252), respectively, did not differ regarding sex, age, prevalence of lifetime MDD, IDS total scores, and IDS symptom clusters (all *p* > .05; see **Table 1**). As expected, participants from MooDFOOD showed significantly higher BMI and WHR than participants from NESDA (all *p* < .05).

**Table 1:** Sample characteristics for the MooDFOOD and NESDA subsamples and the total matched sample.

	Total sample (n = 504)	MooDFOOD (n = 252)	NESDA (n = 252)	Test	p
Sex (*N* female, %)	368 (73.0)	185 (73.4)	183 (72.6)	*χ* ^2^(1) = 0.04	.841
Lifetime MDD (*N*, %)	150 (29.9)	76 (30.4)	74 (29.4)	*χ* ^2^(1) = 0.06	.800
	*M* (SD)	*M* (SD)	*M* (SD)	*F*(1, 502)	
Age	41.93 (13.61)	42.52 (12.85)	41.35 (14.33)	0.93	.335
Body mass index (kg/m^2^)	26.62 (5.33)	31.04 (3.86)	22.19 (1.66)	1,117.92	**<.001**
Waist-to-hip ratio	0.86 (0.09)	0.89 (0.09)	0.82 (0.07)	92.42	**<.001**
IDS total score	13.47 (7.52)	13.69 (7.52)	13.25 (7.53)	0.44	.508
Mood symptoms	4.11 (3.41)	4.13 (3.41)	4.10 (3.41)	0.01	.927
Cognitive symptoms	1.66 (1.67)	1.64 (1.57)	1.69 (1.78)	0.12	.730
Somatic/vegetative symptoms	7.67 (4.03)	7.92 (4.02)	7.41 (4.03)	2.07	.151

Using propensity score matching, samples from the MooDFOOD Trial and the NESDA Study were matched according to sex, age, and distribution of IDS sum scores. Tests for group differences refer to differences between the MooDFOOD and NESDA subsamples, respectively.

M, mean; SD, standard deviation; MDD, major depressive disorder; IDS, Inventory of Depressive Symptomatology Self-Report.

Bold type p < .05.

The total sample comprised *n* = 504 individuals (73.0% women) with a mean age of *M* = 41.93 years (SD = 13.61), a mean BMI of *M* = 26.62 kg/m^2^ (SD = 5.33, range = 18.56–42.10 kg/m^2^), and a mean WHR of *M* = 0.86 (SD = 0.09, range = 0.66–1.12; see **Table 1**). Criteria for a lifetime MDD diagnosis were met by *n* = 150 (29.9%). Based on IDS cutoff scores for clinical severity of depressive symptoms, no values fell in the categories of severe or very severe depressive symptoms. Of the total sample, *n* = 292 (58%) participants had no or low severity of depressive symptoms, *n* = 175 (35%) had mild severity, and *n* = 37 (7%) displayed moderately severe depressive symptoms, respectively.

### Multiple Linear Regression Analyses

Two separate multiple linear regression analyses were calculated to predict BMI and WHR, respectively, based on sociodemographic variables and depressive symptom clusters. The first analysis with BMI as the outcome variable did not yield a significant regression equation: *F*(5, 498) = 2.01, *p* = .076, *R* ^2^ = .02, adjusted *R* ^2^ = .01; see **Table 2** and **Supplementary Figure**). While sociodemographic variables as well as mood and cognitive symptoms were not significantly associated with BMI (all *p* > .05), results showed that the somatic/vegetative symptom cluster had a significant effect (*p* = .013). More somatic/vegetative symptoms were associated with a higher BMI.

**Table 2:** Linear regression analyses of depressive symptom clusters and weight status (*n* = 504; matched sample from MooDFOOD and NESDA)

	Total sample (*N* = 504)			
	*β*	*B*	SE *B*	*p*
Body mass index				
Sex	0.04	-0.46	0.54	.369
Age	0.07	0.03	0.02	.117
Mood symptoms	-0.03	-0.04	0.10	.650
Cognitive symptoms	-0.04	-0.13	0.18	.476
Somatic/vegetative symptoms	0.13	0.18	0.07	**.013**
Waist-to-hip ratio				
Sex	-0.50	-0.10	0.01	**<.001**
Age	0.30	0.00	0.00	**<.001**
Mood symptoms	-0.05	0.00	0.00	.293
Cognitive symptoms	-0.02	0.00	0.00	.704
Somatic/vegetative symptoms	0.12	0.00	0.00	**.006**

Bold type p < .05.

Results of the second analysis with WHR as the outcome variable indicated that there was a collective significant association of the independent variables: *F*(5, 496) = 50.11, *p* < .001, *R* ^2^ = .34, adjusted *R* ^2^ = .33; see **Table 2** and **Supplementary Figure**). Examination of the individual variables showed that sex, age, and somatic/vegetative symptoms yielded significant effects in the model (all *p* < .01). Being male, older age, and more somatic/vegetative symptoms were associated with a higher WHR.

In both models, standardized regression coefficients (beta) for somatic/vegetative symptoms were larger than those for mood and cognitive symptoms, respectively (see **Table 2**).

## Discussion

The aim of this study was to investigate the relationship between depressive symptom clusters (mood symptoms, somatic/vegetative symptoms, and cognitive symptoms) in relation to body weight status, measured by BMI and WHR, in individuals with subthreshold depressive symptoms. Using a sample comprising two large European multicenter studies, the current study is, to our knowledge, the first to address this question.

Results showed that the significant regression model with WHR as the outcome variable yielded a reasonably high model fit, explaining 33% of the variance. It revealed that being male, being of older age, and reporting more depressive symptoms of the somatic/vegetative symptom cluster were significantly associated with higher WHR. Mood and cognitive symptoms did not show any significant contribution. To sum up, age, sex, and somatic/vegetative symptoms, but not mood or cognitive symptoms, were significantly associated with WHR. This finding is in line with previous research indicating that the somatic/affective symptoms of depression rather than the cognitive/affective ones are consistently related to anthropometric measures of obesity (35).

The regression model with BMI as the outcome variable did not reach statistical significance overall, meaning that the majority of the variables did not contribute to BMI. Nevertheless, the factor somatic/vegetative symptoms yielded a significant effect, which points towards the fact that this factor is associated with BMI, but the others do not.

Overall, the results of this study provide further evidence that somatic/vegetative symptoms rather than mood/cognitive symptoms of depression are associated with WHR, and potentially of BMI as well. Somatic/vegetative symptoms include pain, change in appetite and body weight, gastrointestinal, and several arousal-related symptoms (sleep and energy level) (see **Supplementary Table**). The latter are depressive symptoms that are mainly manifested in the somatic area, which might overlap or coincide with complaints and symptoms accompanying or resulting from overweight or obesity and co-occurring somatic conditions.

Moreover and also in line with the international literature, the results suggest that future research should take different body weight status measures (such as WHR) for obesity into account, not only BMI, as other measure index abdominal obesity, which is proposed to be the key factor in the obesity–depression relationship (35, 36).

The current study has important strengths. It included a large, multicenter, multi-country, and propensity score-matched sample and further used standardized assessment instruments. However, there are also some limitations. First, the results might not be generalizable to the full spectrum of depressive disorders and specifically to individuals currently suffering from MDD, as the study population comprised individuals with subthreshold symptoms of depression, but indeed 30% with a lifetime MDD diagnosis. Nevertheless, the full range of body weight state was included in the analysis. Due to the cross-sectional design, causal inferences are not possible and not all potentially relevant control variables (e.g. physical activity) could be included into the analysis. The depressive symptoms being analyzed in this study are administered *via* self-report measures, which might be biased by social desirability and other factors. Also, self-report instruments and clinician-rated scales differ regarding content and weighting of different symptom dimensions. Also, the potential role of certain medical comorbidities regarding this association is not addressed within this analysis.

In conclusion, the present study provides further evidence that there is heterogeneity in depressive symptoms in relation to body weight status, especially as assessed by the WHR. Future studies could investigate the longitudinal course of different depressive symptom clusters and their differential associations with body weight status in the long term. Treatment strategies of both depression and obesity should take the present results into account, e.g., by adapting and targeting interventions to the presented (heterogeneous) symptoms displayed by the individual patient.

## Data Availability Statement

The datasets analyzed in this manuscript are not publicly available. Requests to access the datasets should be directed to www.nesda.nl; www.moodfood-vu.eu.

## Ethics Statement

The studies involving human participants were reviewed and approved by MooDFOOD sample: Research Ethics Committee Govern de les Illes Balears, Palma, Spain (10th of March 2015), the Ethics Committee of the University of Leipzig, Germany (2nd of April 2015), VU Medical Center Amsterdam, the Netherlands (8th of July, 2015) and the NHS National Research Ethics Service (NRES) Committee, SouthWest, UK (Research Ethics Committee number- 15/SW/0153) for University of Exeter (3rd of August 2015). NESDA sample: Ethical Review Board of the VU University Medical Centre. The patients/participants provided their written informed consent to participate in this study.

## Author Contributions

SB and UH were the lead authors on the manuscript and SB and EK analyzed and interpreted the data. MV and IB obtained funding for the MooDFOOD project, designed the MooDFOOD prevention trial, and, together with MC, coordinated the MooDFOOD project. BP, MB, and EW contributed to the design of the MooDFOOD prevention trial. EW led the development and training of the MooDFOOD Food-Related Behavioural Change Intervention. EK and UH coordinated the recruitment, interventions, and follow-ups at the trial center in Germany, University Leipzig. BP and MB coordinated the recruitment, interventions, and follow-ups at the trial center in the Netherlands, VU University Medical Center Amsterdam. EW and MO coordinated the recruitment, interventions, and follow-ups at the trial center in the United Kingdom, University of Exeter. MR and MG coordinated the recruitment, interventions, and follow-ups at the trial center in Spain, University of Balearic Islands. GG set up the logistics for the trial’s data collection. All authors contributed to the writing of the manuscript and approved the final version. Please see www.moodfood-vu.eu for a complete list of the MooDFOOD Prevention Trial Investigators.

## Funding

Funding for this article is provided by the European Union FP7 MooDFOOD Project “Multi-country cOllaborative project on the rOle of Diet, FOodrelated behaviour, and Obesity in the prevention of Depression” (grant agreement no. 613598). This work is supported in the UK by the National Institute for Health Research (NIHR), through the Primary Care Research Network and the NIHR Exeter Clinical Research Facility.

## Conflict of Interest

MR reported receiving grants from the European Union and research funding from Janssen and Lundbeck outside the submitted work. BP reported receiving grants from Janssen Research and Boehringer Ingelheim outside the submitted work. UH reported receiving personal fees from Lundbeck, Janssen Pharmaceutica, Servier, Bayer Pharma, and Medice outside the submitted work.

The remaining authors declare that the research was conducted in the absence of any commercial or financial relationships that could be construed as a potential conflict of interest.

The handling editor declared a shared affiliation, though no other collaboration, with several of the authors SB, NM, ED-S and EK.

## References

[1] World Health Organization (WHO) Depression and other common mental disorders: global health estimates. Geneva: World Health Organization (2017).

[2] Dreimüller N Lieb K Tadić A Engelmann J Wollschläger D Wagner S Body mass index (BMI) in major depressive disorder and its effects on depressive symptomatology and antidepressant response. J Affect Disord (2019) 256:524–31. 10.1016/j.jad.2019.06.067 31280076

[3] Milaneschi Y Simmons WK van Rossum EFC Penninx BWJH Depression and obesity: evidence of shared biological mechanisms. Mol Psychiatry (2019) 24(1):18–33. 10.1038/s41380-018-0017-5 29453413

[4] Gibson-Smith D Bot M Snijder M Nicolaou M Derks EM Stronks K The relation between obesity and depressed mood in a multi-ethnic population. The HELIUS study. Soc Psychiatry Psychiatr Epidemiol (2018) 53(6):629–38. 10.1007/s00127-018-1512-3 PMC5959973 29644388

[5] Luppino FS Wit LM de Bouvy PF Stijnen T Cuijpers P . Overweight, obesity, and depression: a systematic review and meta-analysis of longitudinal studies. Arch Gen Psychiatry (2010) 67(3):220–9. 10.1001/archgenpsychiatry.2010.2 20194822

[6] de Wit L Luppino F van Straten A Penninx B Zitman F Cuijpers P Depression and obesity: a meta-analysis of community-based studies. Psychiatry Res (2010) 178(2):230–5. 10.1016/j.psychres.2009.04.015 20462641

[7] Heo M Pietrobelli A Fontaine KR Sirey JA Faith MS Depressive mood and obesity in US adults: comparison and moderation by sex, age, and race. Int J Obesity (2006) 30(3):513–9. 10.1038/sj.ijo.0803122 16302017

[8] Himmerich H Patsalos O Lichtblau N Ibrahim MAA Dalton B Cytokine research in depression: principles, challenges, and open questions. Front In Psychiatry (2019) 10:30. 10.3389/fpsyt.2019.00030 PMC6374304 30792669

[9] Paans NPG Bot M van Strien T Brouwer IA Visser M Penninx BWJH Eating styles in major depressive disorder: results from a large-scale study. J Psychiatr Res (2018b) 97:38–46. 10.1016/j.jpsychires.2017.11.003 29175296

[10] Penninx BWJH Milaneschi Y Lamers F Vogelzangs N Understanding the somatic consequences of depression: biological mechanisms and the role of depression symptom profile. BMC Med (2013) 11:129. 10.1186/1741-7015-11-129 23672628 PMC3661358

[11] Minkwitz J Scheipl F Cartwright L Campbell IC Chittka T Thormann J Why some obese people become depressed whilst others do not: exploring links between cognitive reactivity, depression and obesity. Psychol Health Med (2019) 24(3):362–73. 10.1080/13548506.2018.1524153 30252503

[12] Hryhorczuk C Sharma S Fulton SE Metabolic disturbances connecting obesity and depression. Front In Neurosci (2013) 7:177. 10.3389/fnins.2013.00177 PMC3791387 24109426

[13] Abdelaal M Le Roux CW Docherty NG Morbidity and mortality associated with obesity. Ann Trans Med (2017) 5(7):161. 10.21037/atm.2017.03.107 PMC5401682 28480197

[14] Nicholson A Kuper H Hemingway H Depression as an aetiologic and prognostic factor in coronary heart disease: a meta-analysis of 6362 events among 146 538 participants in 54 observational studies. Eur Heart J (2006) 27(23):2763–74. 10.1093/eurheartj/ehl338 17082208

[15] Sachs-Ericsson N Burns AB Gordon KH Eckel LA Wonderlich SA Crosby RD Body mass index and depressive symptoms in older adults: the moderating roles of race, sex, and socioeconomic status. Am J Geriatric Psychiatry (2007) 15(9):815–25. 10.1097/JGP.0b013e3180a725d6 17804833

[16] Stunkard AJ Faith MS Allison KC Depression and obesity. Biol Psychiatry (2003) 54(3):330–7. 10.1016/s0006-3223(03)00608-5 12893108

[17] Hach I Ruhl UE Klotsche J Klose M Jacobi F Associations between waist circumference and depressive disorders. J Affect Disord (2006) 92(2–3):305–8. 10.1016/j.jad.2006.01.023 16503357

[18] Ho RCM Niti M Kua EH Ng T-P Body mass index, waist circumference, waist-hip ratio and depressive symptoms in Chinese elderly: a population-based study. Int J Geriatric Psychiatry (2008) 23(4):401–8. 10.1002/gps.1893 17879255

[19] John U Meyer C Rumpf H-J Hapke U Relationships of psychiatric disorders with overweight and obesity in an adult general population. Obesity Res (2005) 13(1):101–9. 10.1038/oby.2005.13 15761168

[20] Paans NPG Bot M Gibson-Smith D van der Does W Spinhoven P Brouwer I The association between personality traits, cognitive reactivity and body mass index is dependent on depressive and/or anxiety status. J Psychosomatic Res (2016) 89:26–31. 10.1016/j.jpsychores.2016.07.013 27663107

[21] Lee C-H Giuliani F The role of inflammation in depression and fatigue. Front In Immunol (2019) 10:1696. 10.3389/fimmu.2019.01696 31379879 PMC6658985

[22] Schmidt FM Sander C Minkwitz J Mergl R Dalton B Holdt LM Serum markers of inflammation mediate the positive association between neuroticism and depression. Front In Psychiatry (2018) 9:609. 10.3389/fpsyt.2018.00609 PMC6256194 30524320

[23] Sullivan PF Neale MC Kendler KS Genetic epidemiology of major depression: review and meta-analysis. Am J Psychiatry (2000) 157(10):1552–62. 10.1176/appi.ajp.157.10.1552 11007705

[24] Robinson MR English G Moser G Lloyd-Jones LR Triplett MA Zhu Z Genotype-covariate interaction effects and the heritability of adult body mass index. Nat Genet (2017) 49(8):1174–81. 10.1038/ng.3912 28692066

[25] Hegerl U Ulke C Fatigue with up- vs downregulated brain arousal should not be confused. Prog In Brain Res (2016) 229:239–54. 10.1016/bs.pbr.2016.06.001 27926440

[26] Hegerl U Largely unnoticed flaws in the fundamentals of depression diagnosis: the semantics of core symptoms. Aust New Z J Psychiatry (2014) 48(12):1166. 10.1177/0004867414559550 25410628

[27] Hegerl U Lam RW Malhi GS McIntyre RS Demyttenaere K Mergl R Conceptualising the neurobiology of fatigue. Aust New Z J Psychiatry (2013) 47(4):312–6. 10.1177/0004867413481505 23568158

[28] Roest AM Thombs BD Grace SL Stewart DE Abbey SE Jonge P de Somatic/affective symptoms, but not cognitive/affective symptoms, of depression after acute coronary syndrome are associated with 12-month all-cause mortality. J Affect Disord (2011) 131(1-3):158–63. 10.1016/j.jad.2010.11.018 21159385

[29] Irvine J Basinski A Baker B Jandciu S Paquette M Cairns J Depression and risk of sudden cardiac death after acute myocardial infarction: testing for the confounding effects of fatigue. Psychosomatic Med (1999) 61(6):729–37. 10.1097/00006842-199911000-00001 10593621

[30] Rush AJ Gullion CM Basco MR Jarrett RB Trivedi MH The Inventory of Depressive Symptomatology (IDS): psychometric properties. Psychol Med (1996) 26(3):477–86. 10.1017/S0033291700035558 8733206

[31] Wardenaar KJ van Veen T Giltay EJ Hollander-Gijsman M Penninx BWJH Zitman FG The structure and dimensionality of the Inventory of Depressive Symptomatology Self Report (IDS-SR) in patients with depressive disorders and healthy controls. J Affect Disord (2010) 125(1-3):146–54. 10.1016/j.jad.2009.12.020 20074811

[32] Gili M Roca M Armengol S Asensio D Garcia-Campayo J Parker G Clinical patterns and treatment outcome in patients with melancholic, atypical and non-melancholic depressions. PloS One (2012) 7(10):e48200. 10.1371/journal.pone.0048200 23110213 PMC3482206

[33] Lamers F Jonge P de Nolen WA Smit JH Zitman FG Beekman ATF Identifying depressive subtypes in a large cohort study: results from the Netherlands Study of Depression and Anxiety (NESDA). J Clin Psychiatry (2010) 71(12):1582–9. 10.4088/JCP.09m05398blu 20673552

[34] Gold PW The organization of the stress system and its dysregulation in depressive illness. Mol Psychiatry (2015) 20S(1):32–47. 10.1038/mp.2014.163 25486982

[35] Wiltink J Michal M Wild PS Zwiener I Blettner M Münzel T Associations between depression and different measures of obesity (BMI, WC, WHtR, WHR). BMC Psychiatry (2013) 13:223. 10.1186/1471-244X-13-223 24028572 PMC3849983

[36] Rivenes AC Harvey SB Mykletun A The relationship between abdominal fat, obesity, and common mental disorders: results from the HUNT study. J Psychosomatic Res (2009) 66(4):269–75. 10.1016/j.jpsychores.2008.07.012 19302883

[37] Després J-P Lemieux I Abdominal obesity and metabolic syndrome. Nature (2006) 444 (7121):881–887. 10.1038/nature05488 17167477

[38] Lamers F Milaneschi Y Jonge P de Giltay EJ Penninx BWJH Metabolic and inflammatory markers: associations with individual depressive symptoms. Psychol Med (2018) 48(7):1102–10. 10.1017/S0033291717002483 28889804

[39] Marijnissen RM Bus BAA Holewijn S Franke B Purandare N Graaf J de Depressive symptom clusters are differentially associated with general and visceral obesity. J Am Geriatrics Soc (2011) 59(1):67–72. 10.1111/j.1532-5415.2010.03228.x 21226677

[40] Roca M Kohls E Gili M Watkins E Owens M Hegerl U Prevention of depression through nutritional strategies in high-risk persons: rationale and design of the MooDFOOD prevention trial. BMC Psychiatry (2016) 16S(1):192. 10.1186/s12888-016-0900-z PMC4898322 27277946

[41] Bot M Brouwer IA Roca M Kohls E Penninx BWJH Watkins E Visser M Effect of multinutrient supplementation and food-related behavioral activation therapy on prevention of major depressive disorder among overweight or obese adults with subsyndromal depressive symptoms: the MooDFOOD randomized clinical trial. JAMA (2019) 321(9):858–868. 10.1001/jama.2019.0556 30835307 PMC6439597

[42] Penninx BWJH Beekman ATF Smit JH Zitman FG Nolen WA Spinhoven P The Netherlands Study of Depression and Anxiety (NESDA): rationale, objectives and methods. Int J Methods In Psychiatr Res (2008) 17(3):121–40. 10.1002/mpr.256 PMC6878352 18763692

[43] Kroenke K Spitzer RL Williams JB The PHQ-9: validity of a brief depression severity measure. J Gen Internal Med (2001) 16(9):606–13. 10.1046/j.1525-1497.2001.016009606.x PMC1495268 11556941

[44] American Psychiatric Association (APA) Diagnostic and Statistical Manual of Mental Disorders (DSM-IV). Washington, DC: APA (2001).

[45] Sheehan DV Lecrubier Y Sheehan KH Amorim P Janavs J Weiller E The Mini-International Neuropsychiatric Interview (M.I.N.I.): the development and validation of a structured diagnostic psychiatric interview for DSM-IV and ICD-10. J Clin Psychiatry (1998) 59 Suppl 20:22–33. 9881538

[46] Wittchen HU Reliability and validity studies of the WHO-Composite International Diagnostic Interview (CIDI): a critical review. J Psychiatr Res (1994) 28(1):57–84. 10.1016/0022-3956(94)90036-1 8064641

[47] Thoemmes F Propensity score matching in SPSS. Hg. v. University of Tübingen. Tübingen: University of Tübingen (2012). Online verfügbar unter http://arxiv.org/ftp/arxiv/papers/1201/1201.6385.pdf, zuletzt geprüft am (accessed 08.08.2019).

[48] Rush AJ Giles DE Schlesser MA Fulton CL Weissenburger J Burns C The inventory for depressive symptomatology (IDS): preliminary findings. Psychiatry Res (1986) 18(1):65–87. 10.1016/0165-1781(86)90060-0 3737788

[49] Paans NPG Bot M Brouwer IA Visser M Roca M Kohls E The association between depression and eating styles in four European countries: the MooDFOOD prevention study. J Psychosomatic Res (2018a) 108S:85–92. 10.1016/j.jpsychores.2018.03.003 29602330

[50] Schaakxs R Comijs HC Lamers F Beekman ATF Penninx BWJH Age-related variability in the presentation of symptoms of major depressive disorder. Psychol Med (2017) 47(3):543–52. 10.1017/S0033291716002579 27786143

[51] Lecrubier Y Sheehan DV Weiller E Amorim P Bonora I Harnett Sheehan K The Mini International Neuropsychiatric Interview (MINI). A short diagnostic structured interview: reliability and validity according to the CIDI. Eur Psychiatry (1997) 12(5):224–31. 10.1016/S0924-9338(97)83296-8

[52] Haro JM Arbabzadeh-Bouchez S Brugha TS Girolamo Gd Guyer ME Jin R Concordance of the Composite International Diagnostic Interview Version 3.0 (CIDI 3.0) with standardized clinical assessments in the WHO World Mental Health surveys. Int J Methods In Psychiatr Res (2006) 15S(4):167–80. 10.1002/mpr.196 PMC6878271 17266013

[53] Trivedi MH Rush AJ Ibrahim HM Carmody TJ Biggs MM Suppes T The Inventory of Depressive Symptomatology, Clinician Rating (IDS-C) and Self-Report (IDS-SR), and the Quick Inventory of Depressive Symptomatology, Clinician Rating (QIDS-C) and Self-Report (QIDS-SR) in public sector patients with mood disorders: a psychometric evaluation. Psychol Med (2004) 34(1):73–82. 10.1017/s0033291703001107 14971628

[54] Cohen J Statistical power analysis for the behavioral sciences. 2nd ed. Hoboken: Taylor and Francis (1988). Online verfügbar unter http://gbv.eblib.com/patron/FullRecord.aspx?p=1192162.

