# Supplementary material for: Depressive Symptom Clusters in Relation to Body Weight Status: Results From Two Large European Multicenter Studies
Source: Front Psychiatry. 2019 Nov 21;10:858. doi: 10.3389/fpsyt.2019.00858 (PMC6882291; doi:10.3389/fpsyt.2019.00858)
Supplement: Supplementary file 1 [file Image_1.pdf]

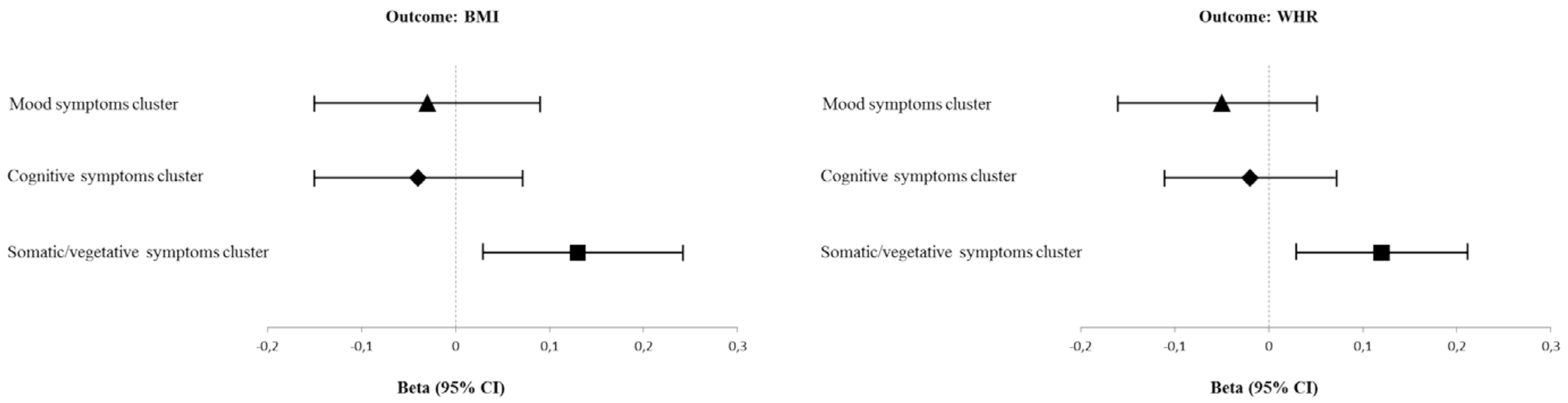


Supplementary Figure

*Linear Regression Analyses of Depressive Symptom Clusters and Weight Status (n = 504; Matched Sample from MooDFOOD and NESDA)*

*Note.* All analyses are adjusted for sex and age. BMI = body mass index; WHR = waist-to-hip ratio; CI = confidence interval.
