# Supplementary material for: Depressive Symptom Clusters in Relation to Body Weight Status: Results From Two Large European Multicenter Studies
Source: Front Psychiatry. 2019 Nov 21;10:858. doi: 10.3389/fpsyt.2019.00858 (PMC6882291; doi:10.3389/fpsyt.2019.00858)
Supplement: Supplementary file 2 [file Table_1.pdf]

Supplementary Table

Items Included in the Depressive Symptom Clusters of the Inventory of Depressive Symptomatology Self Report (IDS-SR30)

Mood Symptoms	*Cognitive Symptoms*	*Somatic/Vegetative Symptoms*
Reduced capacity for pleasure or enjoyment	Concentration/decision-making problems	Aches and pains
Reduced interest in people/ activities	Future pessimism	Appetite increase/decrease
Reduced quality of mood	Self-criticism and blame	Weight increase/decrease
Reduced reactivity of mood	Suicidal thoughts	Diurnal variation with a worse mood in the morning
Feeling anxious or tense		Early morning awakening
Feeling irritable		Low energy level/fatigability
Feeling sad		Other bodily symptoms
Interpersonal sensitivity		Problems falling asleep
Leaden paralysis		Problems sleeping during the night
Panic/phobic symptoms		Sleeping too much
		Psychomotor agitation
		Psychomotor retardation
		Reduced interest in sex
		Constipation/ diarrhea

Note. Depressive symptom clusters according to (Paans et al., 2018a; Paans et al., 2018b; Schaakxs et al., 2017).
